# Two Novel Species of *Cudonia* and *Pleurocordyceps* (Ascomycota) from Mount Tianmu, China

**DOI:** 10.3390/jof12050323

**Published:** 2026-04-28

**Authors:** Yu-Yu Shen, Yan-Jia Chen, Zi-Ruo Deng, Jun-Yin Deng, Chun-Mei Pang, Yao-Bin Song, Ming Dong

**Affiliations:** 1 Key Laboratory of Hangzhou City for Ecosystem Protection and Restoration, Zhejiang Provincial Key Laboratory of Wetland Intelligent Monitoring and Ecological Restoration, College of Life and Environmental Sciences, Hangzhou Normal University, Hangzhou 311121, China; 2024011010002@stu.hznu.edu.cn (Y.-Y.S.); 2024210314018@stu.hznu.edu.cn (Y.-J.C.); 2024210314021@stu.hznu.edu.cn (Z.-R.D.); jydeng@hznu.edu.cn (J.-Y.D.); dongming@hznu.edu.cn (M.D.); 2 Management Bureau of Tianmu Mountain National Nature Reserve, Hangzhou 311311, China; 13868013206@163.com

**Keywords:** entomopathogenic fungi, morphology, multigene fragment, phylogeny, taxonomy

## Abstract

*Cudonia* and *Pleurocordyceps* comprise a relatively limited number of species worldwide. In particular, *Pleurocordyceps* represents an ecologically important group of entomopathogenic fungi with considerable potential for medicinal applications. In this study, two novel species, *Cudonia aurantiaca* and *Pleurocordyceps tianmushanensis*, were collected from the National Nature Reserve of Mount Tianmu, Zhejiang Province, China. Detailed morphological descriptions and illustrations are provided, and genus-level phylogenetic relationships are inferred based on a combined multi-locus sequence dataset comprising the internal transcribed spacer (ITS), small subunit ribosomal RNA (SSU), large subunit ribosomal RNA (LSU), translation elongation factor 1-alpha (*tef-1α*), RNA polymerase II largest subunit (*rpb1*), and RNA polymerase II second largest subunit (*rpb2*) gene regions. This study contributes to a better understanding of the diversity and taxonomy of *Cudonia* and *Pleurocordyceps.*

## 1. Introduction

*Cudonia* belongs to Cudoniaceae, Rhytismatales, Leotiomycetes, and was established by Fries in 1849, with the type species *C. circinans* [1]. Initially, *Cudonia* was placed in Geoglossaceae based on its rounded to clavate ascomata and cylindrical stipe [2,3,4]. The asci are clavate, J-, 8-spored. Paraphyses are filiform, curved to circinate, or occasionally straight distally. Ascospores are filiform to acicular, becoming one- to multiseptate at maturity [3,4]. However, because morphological differences among species are subtle, the systematic position of the genus remained controversial in early taxonomic treatments [2,3,4,5,6,7,8,9,10]. With advances in molecular phylogenetics, the taxonomic placement of *Cudonia* has received increasing research attention [11,12,13,14,15]. In 2001, Kirk et al. [16] established the family Cudoniaceae (Helotiales, Ascomycotina), with *Cudonia* as the type genus. Furthermore, molecular phylogenetics has confirmed that Cudoniaceae should be placed in Rhytismatales [3,17].

Species of *Cudonia* are widely distributed in temperate regions, with a few species extending into subtropical zones, and they typically occur in coniferous forests or on humus layers associated with mosses [3]. To date, 19 species of *Cudonia* have been recorded in Index Fungorum (https://indexfungorum.org/Names/Names.asp, accessed 20 March 2026). Notably, several *Cudonia* species lack molecular data, which hinders comprehensive taxonomic and phylogenetic studies of the genus [15].

*Pleurocordyceps* is placed in Polycephalomycetaceae, Hypocreales, Sordariomycetes, and was established by Wang et al. [18] with the type species *P. sinensis*. *Pleurocordyceps* is the most species-rich genus within the family Polycephalomycetaceae, with 25 species currently recorded in Index Fungorum (https://indexfungorum.org/Names/Names.asp, accessed 20 March 2026) [18,19]. In China, 15 species of *Pleurocordyceps* have been reported [19,20]. The genus is distinguished from closely related taxa by the presence of laterally fertile, pulvinate stromata near the apex of the sexual morph, as well as by the occurrence of two distinct types of phialides and conidia in the asexual morph [18,19,20,21]. Species of *Pleurocordyceps* have been reported from a wide range of insect hosts, including members of Coleoptera, Hymenoptera, Hemiptera, Lepidoptera, Orthoptera, and Homoptera [11,22,23]. In addition to parasitizing insects, most species in the genus are also parasites of fungi [20,21,24]. Species of *Pleurocordyceps* exhibit considerable potential for the production of diverse secondary metabolites [21,24].

Over the past decades, several novel species of *Cudonia* and *Pleurocordyceps* have been described [4,15,19,20,21]. Nevertheless, a portion of the species diversity within both genera remains undocumented [4,25]. In this study, we describe two novel species, *Cudonia aurantiaca* and *Pleurocordyceps tianmushanensis*, collected from the National Nature Reserve of Mount Tianmu, Zhejiang Province, China. Detailed morphological descriptions and phylogenetic analyses are provided to support their taxonomic placement.

## 2. Materials and Methods

### 2.1. Sample Collection, Isolation and Morphological Studies

Fresh specimens were collected from the National Nature Reserve of Mount Tianmu, Zhejiang Province, China, during the period 2023–2025. For *Pleurocordyceps* species, one fresh sample was collected, and more than ten samples of *Cudonia* species were collected from the same locality. The elevation of the reserve ranges from approximately 300 to 1500 m, with abundant vegetation. Vegetation types include mixed coniferous broadleaved forest (300–1200 m, a.s.l.), mixed evergreen deciduous broadleaved forest (800–1100 m, a.s.l.), and deciduous broadleaved forest (1100–1500 m, a.s.l.). The reserve has an average annual temperature range from 8.8 to 14.8 °C, with annual precipitation between 1390 and 1870 mm [26].

Between June and August 2025, for *Pleurocordyceps* species, strains were isolated from fresh specimens by aseptically excising small pieces of mycelium from the host and transferring them onto PDA plates using a sterile needle. The cultures were incubated at 25 °C until pure cultures were obtained. The pure culture was deposited in the Herbarium of Hangzhou Normal University (HTC). The strain of *Cudonia* species was not isolated. All voucher specimens are stored in HTC.

The macroscopic characteristics and microstructure are described for two samples of *Cudonia*, one sample and two strains of *Pleurocordyceps* species. Micromorphological observations were performed using a super depth-of-field microscope (KEYENCE VHX-6000, Osaka, Japan) and a compound microscope (Eclipse Ni, Belfast, UK). The colors of the specimens were coded according to Kornerup & Wanscher [27]. For *Cudonia* species, microscopic structures were obtained directly from free-hand sections of the dried ascomata and observed in Lactophenol Cotton Blue (LPCB). For *Pleurocordyceps* species, fresh strain samples were observed in LPCB as well. The morphological structures were measured with 20 measurements using ImageJ (v.1.54m, National Institutes of Health, Bethesda, MD, USA). Ranges in the measurements of microcharacters are presented as ranges from the minimum to the maximum values. The photographic plates were processed using Adobe Photoshop CC 2020 (Adobe Systems, San Jose, CA, USA).

### 2.2. DNA Extraction, PCR Amplification and Sequencing

Total DNA was extracted from ascomata and strains using the Ezup Column Fungal Genomic DNA Extraction Kit (Sangon Co., Ltd., Shanghai, China), following the manufacturer’s instructions. ITS, SSU, LSU, *rpb1*, *rpb2*, and *tef-1α* gene amplifications were performed using the ITS1/ITS4, NS1/NS4, LR0R/LR5, CRPB1A/RPB1Cr, fRPB25F/fRPB2-7Cr, and 983F/2218R primers, respectively [28,29,30,31,32,33,34]. The nuclear gene amplification reactions followed the protocol outlined by Yang et al. [35]. The PCR products were sequenced by Sangon Biotech (Shanghai, China). All newly generated sequences were assembled using SeqMan. Sequence quality was assessed using BioEdit (v.7.7.1) and BLAST searches following the procedures described by Nilsson et al. [36]. The new sequences were uploaded to GenBank, and accession numbers were assigned (Table 1 and Table 2).

### 2.3. Phylogenetic Analyses

Reference taxa for phylogenetic analyses were selected based on BLAST search results (https://blast.ncbi.nlm.nih.gov/Blast.cgi, accessed 20 March 2026) and previously published datasets [11,15,19]. Individual loci were aligned using MAFFT v.7 (https://mafft.cbrc.jp/alignment/server, accessed 20 March 2026) and manually adjusted and trimmed in BioEdit [37]. Phylogenetic relationships were inferred using maximum likelihood (ML) and Bayesian inference (BI) analyses. ML analysis was conducted using RAxML-HPC2 v.8.2.12 on the CIPRES Science Gateway web server (http://www.phylo.org/portal2, accessed 20 March 2026), employing 1000 rapid bootstrap replicates and the GTRGAMMA+I model. For the ML analysis, bootstrap support values of 75% or higher were noted above the nodes in the phylogenetic tree. BI phylogenetic analyses were determined by Markov Chain Monte Carlo (MCMC) sampling using MrBayes on ACCESS via the CIPRES science gateway (http://www.phylo.org, accessed 20 March 2026). BI analyses were performed with six independent MCMC runs in our operation, and trees were sampled every 100 generations. The analyses were stopped after 5,000,000 generations when the average standard deviation of split frequencies was below 0.01. The first 25% of the resulting trees were discarded as burn-in and posterior probabilities (PP) were calculated from the remaining sampled trees. Outgroup taxa were selected following previous related studies [15,19]. The ML and Bayesian trees were visualized with FigTree v.1.4.0 (http://tree.bio.ed.ac.uk/software/figtree/, accessed 20 March 2026).

## 3. Results

### 3.1. Phylogenetics

**Figure 1:**
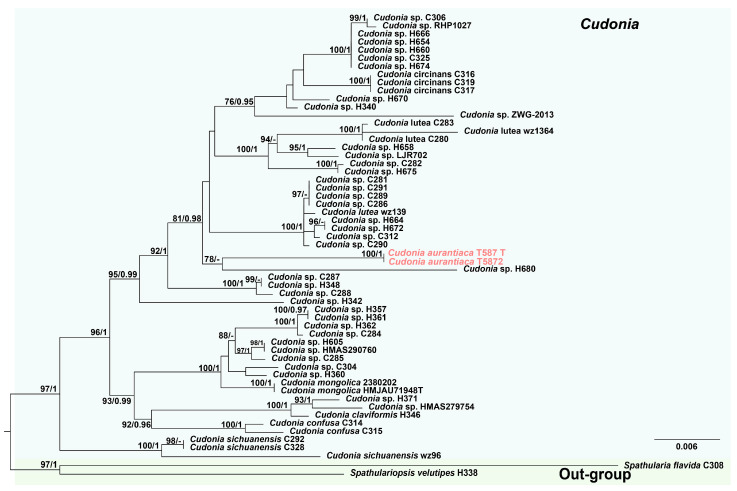
ML phylogenetic tree of *Cudonia* combining ITS, LSU, *rpb2* and *tef-1α* sequence data. The tree is rooted to *Spathulariopsis velutipes* (H338) and *Spathularia flavida* (C308). ML bootstrap values ≥ 75% and PP from Bayesian Inference ≥ 0.95 are given above branches. *Cudonia aurantiaca* is indicated in pink. The taxa marked with a “T” are type specimens. *Cudonia* is highlighted in light blue, while the outgroup is marked with a light green background.

**Figure 2:**
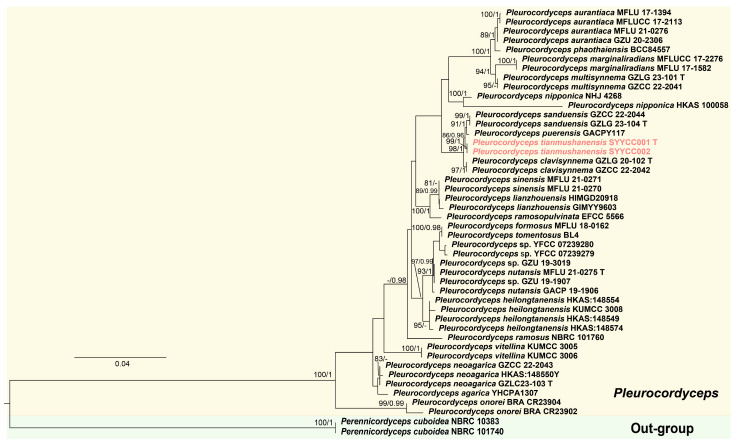
Phylogram of the *Pleurocordyceps* clade generated by Maximum Likelihood (ML) analysis of ITS, LSU, SSU, *tef-1α*, *rpb1*, and *rpb2* sequence data, showing the position of *P. tianmushanensis* (indicated in pink). The tree is rooted to *Perennicordyceps cuboidea* (NBRC 101740) and *Pe. cuboidea* (NBRC 10383). ML bootstrap values ≥ 75% and PP from Bayesian Inference ≥ 0.95 are given above branches. The taxa marked with a “T” are type specimens. *Pleurocordyceps* is highlighted in light yellow, while the outgroup is marked with a light green background.

As the topologies of ML and BI trees were similar, and only the ML tree is presented here (Figure 1 and Figure 2). Phylogenetic analyses of *Cudonia* species were conducted based on a combined dataset of ITS, LSU, *rpb2*, and *tef-1α* sequences from 55 samples representing 7 *Cudonia* species. The 55 samples of *Cudonia,* which included two newly generated sequences of *Cudonia* (T587 and T5872), formed a distinct clade with strong statistical support (BS = 97%, PP = 1; Figure 1). Samples of *C. aurantiaca* formed a distinct clade with strong statistical support (BS = 100%, PP = 1). *Cudonia aurantiaca* was recovered as sister to *Cudonia* sp. (H680) with low bootstrap support (BS = 78%, PP = 0.94). In addition, *C. aurantiaca* clustered with *C. lutea* in a well-supported lineage (BS = 81%, PP = 0.98).

Phylogenetic trees of *Pleurocordyceps* were constructed using a combined six-gene dataset, including ITS, SSU, LSU, *rpb1*, *rpb2* and *tef-1α* sequence data from 45 samples representing 22 *Pleurocordyceps* species. In the phylogenetic analysis of *Pleurocordyceps*, the samples (SYYCC001 and SYYCC002) formed a distinct and well-supported lineage (BS = 99%, PP = 1; Figure 2). *Pleurocordyceps tianmushanensis* was recovered as sister to *P. sanduensis* and *P. puerensis* with strong statistical support (BS = 86%, PP = 0.96). In addition, *Pleurocordyceps tianmushanensis*, *P. sanduensis*, *P. puerensis*, and *P. clavisynnema* formed a distinct clade with strong statistical support (BS = 98%, PP = 1; Figure 2).

### 3.2. Taxonomy

**Figure 3:**
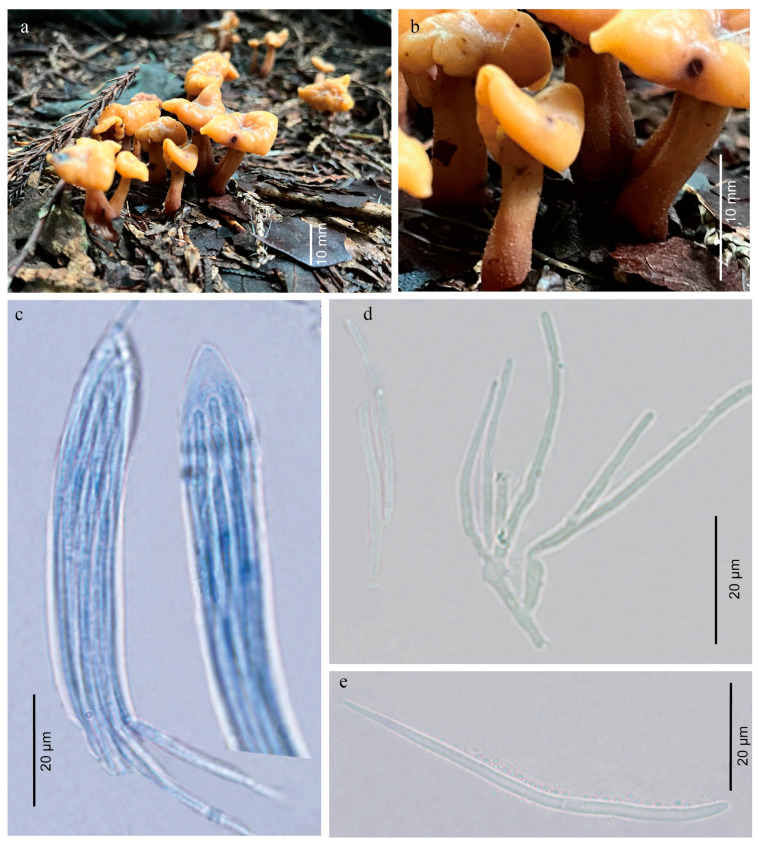
(**a**) Habitat of *Cudonia aurantiaca*. (**b**) Surface of sipe. (**c**) Asci and ascospores. (**d**) Paraphyses. (**e**) Conidia and one ascospore producing conidia.

**Figure 4:**
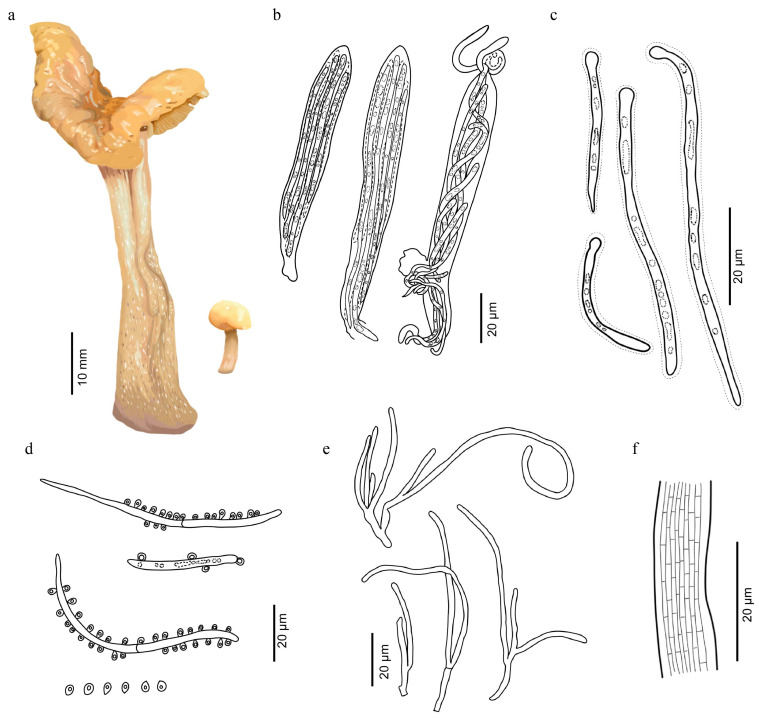
(**a**) Drawing of ascomata. (**b**) Asci and ascospores. (**c**) Ascospores. (**d**) Conidia and three ascospores producing conidia. (**e**) Paraphyses. (**f**) Longitudinal section of the stipe on its surface.

***Cudonia aurantiaca*** Y.Y. Shen & Y.B. Song, sp. nov. Figure 3 and Figure 4.

Index Fungorum number: IF905160; Facesoffungi number: FoF 19637.

Etymology: The specific epithet “aurantiaca” refers to the orange color of the ascomata.

Holotype: HTC (collecting numbers T587), China, Zhejiang Province, Hangzhou, National Nature Reserve of Mount Tianmu at 1176 m a.s.l., 30.34 N, 119.43 E, 25 September 2023, Yu-Yu Shen. Scattered in litter under mixed conifer-broadleaf forest. GenBank accessions: PZ070749 (ITS), PZ125084 (LSU), PZ071831 (SSU), PZ124377 (*rpb2*), and PZ124383 (*tef-1α*).

Diagnosis: *Cudonia aurantiaca* is somewhat similar to *C. mongolica*. However, *C*. *aurantiaca* has a darker color in the saddle-shaped pileus and a darker color in the stipes, covered with obvious and small white spines on the surface of the stipe.

Description: Ascomata gregarious or cespitose, saddle-shaped, stipitate, the edge rolling toward the stipe, 15.3–57.2 mm high. Ascigerous portion capitate, semiorbicular, 11.2–57.2 mm diam. When young, the surface of hymenium light orange to golden yellow (5A4-5A6), greyish orange (5B5-5B6) to brownish orange (5C5-5C6) when old, smooth. Receptaculum pale orange to light orange (5A2-5A5). Stipe 11.1–17.4 × 4.7–74.4 mm, subcylindrical, slightly swollen base, greyish orange (5B4-5B5) when young, then pale orange to light orange (5A2-5A5) with growing, smooth or apical part with longitudinal striations, covered with obvious small white spines.

Hymenium about 200 μm thick, consisting of paraphyses and asci at different stages of development. Asci clavate, 88.1–187.7 × 9.5–17.5 μm (x = 101.6 × 12.8 μm, n = 20), clavate to narrowly clavate, attenuated towards the base, 8-spored, apical portion narrowly round, J-, croziers present. Ascospores 39.5–89.6 × 1.4–3.2 μm (x = 69.7 × 2.3 μm, n = 20), clavate-filiform to acicular, colorless and hyaline, thin-walled, round at apex, acuminate at base, multiguttulate, non-septate, the wall with a gelatinous layer 1–2 μm thick. Ascoconidia 1.1–2.4 × 1.1–2.1 μm (x = 1.7 × 1.5 μm, n = 20), subglobose to obovoid, smooth, thin-walled, 1-celled, 1-guttulate, colorless and hyaline, sometimes nearly filling the asci. Paraphyses filiform, often branched and anastomosing below, strongly curved to circinate above, lower portion about 2.5 μm in diam, apical portion about 1.5 μm in diam.

Habitat and distribution: Scattered in litter. The fungus is currently known only from its type locality.

Additional material examined: HTC (collecting numbers T5872, paratype), China, Zhejiang Province, Hangzhou, the National Nature Reserve of Mount Tianmu at 1176 m a.s.l., 30.34 N, 119.43 E, 25 September 2023, Yu-Yu Shen. GenBank accessions: PZ070750 (ITS), PZ145085 (LSU), PZ071832 (SSU), PZ124378 (*rpb2*), and PZ124384 (*tef-1α*).

Notes: *Cudonia aurantiaca* is characterized by saddle-shaped ascomata with cylindrical stipes; asci are clavate, J-, 8-spored, ascospores are filiform to acicular, hyaline, one- to several-septate at maturity, and surrounded by a gelatinous sheath. These characteristics are consistent with the diagnostic features of the genus *Cudonia* [3,4,15]. *Cudonia aurantiaca* and *C. circinans* (type species of *Cudonia*) can be distinguished by ascocarps and stipe characteristics. Ascocarps of *C. circinans* are cream to dark brown, with striate to ridged and drab to dark brown stipe [5]. *Cudonia aurantiaca* has saddle-shaped and orange pileus, and greyish orange stipes, especially covered with obvious and small white spines on the surface of the stipe.

According to our phylogenetic analyses, samples of *C*. *aurantiaca* are grouped into a single clade with maximum statistical support (BS = 100%, PP = 1, Figure 1) based on ITS, LSU, *rpb2*, and *tef-1α* sequence data. *Cudonia aurantiaca* clustered with *C. lutea* in a well-supported lineage (BS = 81%, PP = 0.98). Base pair differences with gaps between *C. aurantiaca* and *C. lutea* (wz 139) are 7/508 (1.4%) in ITS, 10/799 (1.3%) in LSU. Morphologically, *C. lutea* and *C. aurantiaca* are similar, but *C. lutea* differs in having longer and narrower ascospores (75–80 × 1.5 μm) compared with those of *C. aurantiaca* (69.7 × 2.3 μm) [38]. Although *C. aurantiaca* is phylogenetically distant from *C. mongolica*, the two species share several morphological similarities. Compared with *C. mongolica*, *C. aurantiaca* is distinguished by its saddle-shaped ascigerous portion. In addition, *C. mongolica* is characterized by the presence of small crystals on the surface of the stipe, but *C. aurantiaca* is covered with obvious and small white spines [15]. Hence, this study introduces *C. aurantiaca* as a novel species based on morphological and phylogenetic analyses.

**Figure 5:**
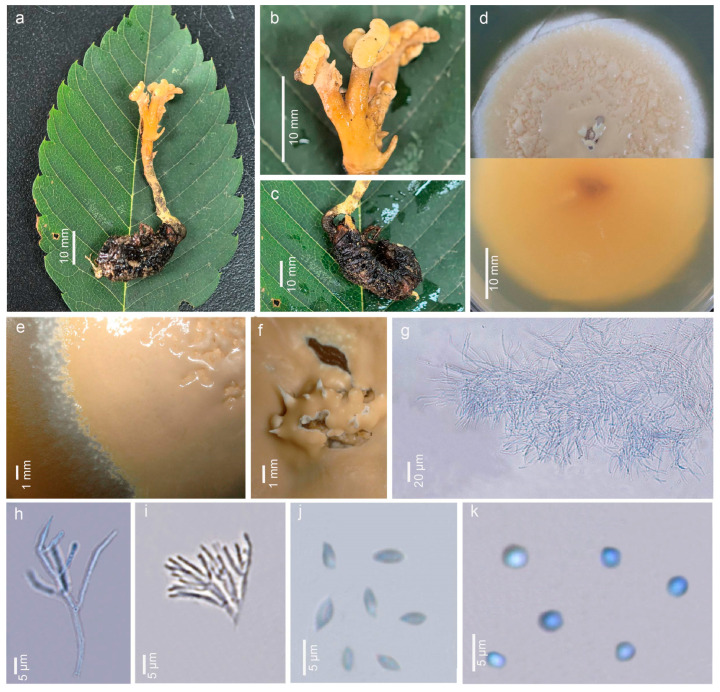
*Pleurocordyceps tianmushanensis* (SYYCC001, Holotype). (**a**) Overview of *P. tianmushanensis*. (**b**) Synnemata on the host. (**c**) Host of *P. tianmushanensis*. (**d**) Upper and back sides of the colony on PDA medium. (**e**,**f**) Conidial masses on the culture. (**g**) Conidiophores. (**h**) α-phialides. (**i**) β-phialides. (**j**) α-conidia. (**k**) β-conidia.

***Pleurocordyceps tianmushanensis*** Y.Y. Shen & Y.B. Song, sp. nov. Figure 5.

Index Fungorum number: IF905161; Facesoffungi number: FoF 19638.

Etymology: The specific epithet “tianmushanensis” refers to the locality where the species was collected, Mount Tianmu, Zhejiang Province, China.

Holotype: HTC (collecting numbers: SYYCC001), China, Zhejiang Province, Hangzhou, National Nature Reserve of Mount Tianmu at 300 m a.s.l., 30.32 N, 119.45 E. Parasitic on larvae of Scarabaeidae species (Coleoptera), grown in soil under mixed conifer-broadleaf forest, collected on 18 June 2025, Zi-Wen Zhang. GenBank accessions: PZ070746 (ITS), PZ070751 (LSU), PZ134459 (SSU), PZ124379 (*rpb1*), PZ095765 (*rpb2*), and PZ124381 (*tef-1α*).

Description: Sexual morph: Not observed. Host 21.90 × 1.8–4.48 mm, a larva of Scarabaeidae species (Coleoptera), characteristically C-shaped, brown. Stromata arising from the head of the host, branched, cylindrical, light yellow to yellow (5A8), 41.05 × 2.10–4.10 mm, with sterile apical. Stipes 39.05 × 2.10–4.10 mm, clavate, the color gradually becoming lighter towards the apex, light yellow to orange (4A7), with hemispherical, cap-shaped parts (4.76–5.24 × 1.52–1.71 mm). Asexual morph: Hyphomycetous. Culture characteristics: Colonies on PDA fast-growing, obtained from tissue, reaching 5 cm wide in 15 days at 28 °C, white, reverse yellow (5A7) to brown (5C8), presenting multiple radiating ring-like patterns. Synnemata emerging after 20 days, solitary, unbranched, 0.2–0.6 mm long, distributed at the edge, with small or without a fertile head. Conidial masses covered the surface of the colony, pale yellow (4A3) when young, later change to brown color (4C4), slime. Conidiophore 21–42 μm long (x = 31.4 μm, n = 30). Phialides exist in α-phialides and β-phialides. α-phialides 18.6–59.3 × 0.7–2.4 μm (x = 34.6 × 1.4 μm, n = 20), smooth, hyaline, solitary. β-phialides 25.0–84.7 × 0.9–2.9 μm (x = 47.0 × 1.6 μm, n = 20), smooth, hyaline, solitary. α-conidia 0.8–2.1 μm (x = 1.4 μm, n = 30) wide, globose, unicellular, smooth-walled; β-conidia 1.7–3.5 × 0.8–1.6 μm (x = 2.7 × 1.3 μm, n = 30) fusiform, unicellular, hyaline, smooth-walled.

Habitat and distribution: Scattered in soil. The fungus is currently known only from its type locality.

Material examined: China, Zhejiang Province, Hangzhou, the National Nature Reserve of Mount Tianmu. Parasitic on larvae of Scarabaeidae species (Coleoptera), in soil, collected on 18 June 2025, Zi-Weng Zhang, CC001 (HTC strain numbers: SYYCC001). Ex-type living culture, HTC (strain numbers: SYYCC002). GenBank accessions (SYYCC002): PZ070747 (ITS), PZ070752 (LSU), PZ134460 (SSU), PZ124380 (*rpb1*), PZ095766 (*rpb2*), and PZ124382 (*tef-1α*).

Notes: *Pleurocordyceps tianmushanensis* is distinguished by the presence of two types of phialides and conidia in the asexual morph, a key diagnostic feature shared with other species of *Pleurocordyceps* [21]. *Pleurocordyceps tianmushanensis* and *P. sinensis* (type species of *Pleurocordyceps*) are differentiated by synnemata. Synnemata of *P. sinensis* were bigger compared to *P. tianmushanensis* (50–60 vs. 2–10 mm, respectively), clavate and capitate [18].

According to our phylogenetic analyses, samples of *Pleurocordyceps tianmushanensis* are sister to *P. sanduensis* and *P. puerensis* with high statistical support (BS = 92%, PP = 1, Figure 2). Samples of *Pleurocordyceps tianmushanensis*, *P. sanduensis*, *P. puerensis* and *P. clavisynnema* formed a distinct clade with strong statistical support (BS = 86%, PP = 0.96; Figure 2). The four species are closed in phylogeny. Base pair differences with gaps between *P. tianmushanensis* and *P. sanduensis* are 1/607 (0.2%) in ITS, 0/734 in LSU, 7/1082 (0.6%) in SSU, 7/846 (0.8%) in *tef-1α*, and 4/918 (0.4%) in *rpb2* (Table 3). Between *P. tianmushanensis* and *P. puerensis*, the differences are 4/512 (0.8%) in ITS, 0/716 in LSU, 15/798 (1.9%) in SSU, 5/855 (0.6%) in *tef-1α*, and 1/594 (0.2%) in *rpb1* [25]. Base pair differences between *P. tianmushanensis* and *P. clavisynnema* are 1/594 (0.2%) in ITS, 0/1231 in SSU, 0/856 in LSU, 2/642 (0.3%) in *rpb1*, 4/968 (0.4%) in *rpb2*, and 4/878 (0.5%) in *tef-1α*. We further compared the base pair differences with gaps among the other three species (*P. sanduensis*, *P. puerensis*, and *P. clavisynnema*), and the results show that the differences among these species are small, especially for the ITS and LSU (Table 3). Specifically, *Pleurocordyceps sanduensis* differs from *P. clavisynnema* by 0/518 in ITS, 0/815 in LSU, 8/1130 (0.3%) in SSU, 2/678 (0.3%) in *rpb1*, 3/1050 (0.3%) in *rpb2,* and 23/824 (2.8%) in *tef-1α* [18,19]. *Pleurocordyceps sanduensis* differs from *P. puerensis* by 2 bp in ITS, 0 bp in LSU, 1 bp in *rpb1*, and 6 bp in *tef-1α*. Between *P. clavisynnema* and *P. puerensis*, the differences are 3/552 (0.5%) in ITS, 0/764 in LSU, 0/800 in SSU, 1/676 (0.1%) in *rpb1*, and 7/795 (0.9%) in *tef-1α* [19,25]. *Pleurocordyceps tianmushanensis* was isolated from scarabaeid larvae, while *P. sanduensis* and *P. clavisynnema* parasites on *Ophiocordyceps neogryllotalpae*; *Pleurocordyceps puerensis* parasites on larvae of Coleoptera. Morphologically, *P. tianmushanensis* differs from *P. sanduensis* in larger phialides (α-phialides: 34.56 × 1.41 vs. 14.1 × 1.45 μm; β-phialides: 47.0 × 1.6 vs. 26.2 × 1.35 μm, respectively), and smaller conidia (α-conidia: 1.4 vs. 2.6 μm; β-conidia: 2.7 × 1.3 vs. 4 × 1.8 μm, respectively) [19,25]. *Pleurocordyceps tianmushanensis* differs from *P. puerensis* in orange stipe [25]. Due to the lack of sexual morphology of *P. tianmushanensis*, it is impossible to compare *P. tianmushanensis* and *P. puerensis* in morphological characteristics [25]. Hence, this study introduces *P. tianmushanensis* as a novel species based on morphological and phylogenetic analyses.

## 4. Discussion

We described two novel species, *Cudonia aurantiaca* and *Pleurocordyceps tianmushanensis*, using morphological observations and phylogenetic analyses. *Cudonia aurantiaca* is characterized by saddle-shaped ascomata with cylindrical stipes; asci are clavate, J-, 8-spored, consistent with the diagnostic features of the genus *Cudonia* [3,4,15]. Two novel species were collected from the National Nature Reserve of Mount Tianmu, Zhejiang Province, China. *Cudonia* species typically occur in humus layers and are widely distributed in temperate regions, with a few species in subtropical zones [8]. Consistent with this pattern, *Cudonia aurantiaca* is scattered in litter under mixed conifer-broadleaf forest in subtropical zones. *Pleurocordyceps tianmushanensis* grown in soil under mixed conifer-broadleaf forest as well. *Pleurocordyceps* species has a wide range of hosts, including insects (e.g., species of Coleoptera, Hymenoptera, Hemiptera, Lepidoptera, Orthoptera, and Homoptera) and fungi (e.g., species of *Ophiocordyceps*) [20]. Consistently, *Pleurocordyceps tianmushanensis* parasitized on larvae of Coleoptera species. The extensive host ranges and distinctive biogeographic distributions of *Pleurocordyceps* further reflect their high ecological adaptability. In addition, species of *Pleurocordyceps* exhibit considerable potential for the production of diverse secondary metabolites. For example, *P. nipponicus* and *P. phaothaiensis* have been reported to produce natural compounds with antioxidant, antibacterial, antitumorigenic, anti-inflammatory, and antimicrobial activities [20,21,28]. Despite these findings, substantial knowledge gaps remain regarding the chemistry, industrial potential, and ecological roles of *Pleurocordyceps* species [15]. Therefore, further investigations are warranted to comprehensively explore the diversity, functions, and potential applications of secondary metabolites within this genus.

Based on integrative taxonomic and molecular phylogenetic evidence, this study adds one novel species each to the genera *Cudonia* and *Pleurocordyceps* from China, thereby expanding their known geographical diversity and providing a valuable basis for future studies on the evolution and utilization of these genera.

## Figures and Tables

**Table 1:** Sequence information and GenBank numbers of samples used in *Cudonia* study.

Species	Voucher	ITS	LSU	*rpb2*	*tef-1α*	Country
*Cudonia claviformis*	H346	KC833169	KC833213	KC833298	KC833380	China
***C. aurantiaca*** T	**T587**	**PZ070749**	**PZ125084**	**PZ124377**	**PZ124383**	**China**
***C. aurantiaca***	**T5872**	**PZ070750**	**PZ125085**	**PZ124378**	**PZ124384**	**China**
*C. confusa*	C314	KC833165	KC833216	KC833300	KC833383	Finland
*C. confusa*	C315	KC833166	KC833217	-	KC833384	Finland
*C. lutea*	C280	KC833150	KC833186	KC833279	KC833354	China
*C. lutea*	C283	KC833151	KC833187	KC833280	KC833353	China
*C. lutea*	wz1364	AF433149	AF433138	-	-	-
*C. lutea*	wz139	AF433151	AF433139	-	-	-
*C. mongolica*	2380202	PQ045732	PQ045734	PQ057765	PQ057770	-
*C. mongolica* T	HMJAU71948	PQ045731	PQ045733	PQ057764	PQ057769	-
*C. sichuanensis*	C292	KC833121	KC833218	KC833301	KC833385	China
*C. sichuanensis*	C328	KC833122	KC833220	KC833302	KC833386	China
*C. sichuanensis*	wz96	AF433148	AF433136	-	-	-
*C. circinans*	C316	KC833156	KC833182	KC833275	KC833349	Switzerland
*C. circinans*	C317	KC833157	KC833183	KC833276	KC833350	Switzerland
*C. circinans*	C319	KC833158	KC833184	KC833277	KC833351	Switzerland
*Cudonia* sp.	C281	KC833146	KC833191	KC833284	KC833358	China
*Cudonia* sp.	C282	KC833148	KC833188	KC833281	KC833355	China
*Cudonia* sp.	C284	KC833129	KC833208	KC833323	KC833375	China
*Cudonia* sp.	C285	KC833127	KC833209	KC833324	KC833376	China
*Cudonia* sp.	C286	KC833139	KC833195	KC833287	KC833362	China
*Cudonia* sp.	C287	KC833134	KC833201	KC833294	KC833368	China
*Cudonia* sp.	C288	KC833135	KC833202	KC833295	KC833370	China
*Cudonia* sp.	C289	KC833140	KC833196	KC833288	KC833363	China
*Cudonia* sp.	C290	KC833141	KC833197	KC833289	KC833364	China
*Cudonia* sp.	C291	KC833142	KC833198	KC833290	KC833365	China
*Cudonia* sp.	C304	KC833125	KC833212	KC833320	KC833379	China
*Cudonia* sp.	C306	KC833163	KC833175	KC833269	KC833343	China
*Cudonia* sp.	C312	KC833143	KC833194	KC833291	KC833361	China
*Cudonia* sp.	C325	KC833159	KC833180	KC833273	-	China
*Cudonia* sp.	H340	KC833155	KC833181	KC833274	KC833348	USA
*Cudonia* sp.	H342	KC833133	KC833204	KC833297	KC833371	USA
*Cudonia* sp.	H348	KC833136	KC833203	KC833296	KC833369	China
*Cudonia* sp.	H357	KC833131	KC833205	KC833325	KC833374	China
*Cudonia* sp.	H360	KC833126	KC833211	-	KC833378	China
*Cudonia* sp.	H361	KC833132	KC833206	-	KC833372	China
*Cudonia* sp.	H362	KC833130	KC833207	KC833321	KC833373	China
*Cudonia* sp.	H371	KC833124	KC833214	KC833299	KC833381	China
*Cudonia* sp.	H605	KC833128	KC833210	KC833322	KC833377	China
*Cudonia* sp.	H654	KC833160	KC833179	KC833272	KC833347	China
*Cudonia* sp.	H658	KC833147	KC833190	KC833283	KC833357	China
*Cudonia* sp.	H660	KC833161	KC833178	KC833271	KC833346	China
*Cudonia* sp.	H664	KC833144	KC833193	KC833286	KC833360	China
*Cudonia* sp.	H666	KC833162	-	KC833270	KC833345	China
*Cudonia* sp.	H670	KC833152	KC833185	KC833278	KC833352	China
*Cudonia* sp.	H672	KC833145	KC833192	KC833285	KC833359	China
*Cudonia* sp.	H674	KC833164	KC833176	-	KC833344	China
*Cudonia* sp.	H675	KC833149	KC833189	KC833282	KC833356	China
*Cudonia* sp.	H680	KC833137	KC833199	KC833292	KC833366	China
*Cudonia* sp.	HMAS279754	MT605576	MT605578	MT624734	MT622853	-
*Cudonia* sp.	HMAS290760	MT605577	MT605579	MT624735	MT622854	-
*Cudonia* sp.	LJR702	PP853393	PP851390	-	-	China
*Cudonia* sp.	RHP1027	KC833173	KC833268	-	KC833434	China
*Cudonia* sp.	ZWG-2013	KC833174	-	KC833304	KC833435	USA
*Spathularia flavida*	C308	KC833105	KC833250	KC833334	KC833418	China
*Spathulariopsis velutipes*	H338	KC833089	KC833240	KC833327	KC833408	USA

Notes: Newly generated sequences are indicated in bold. “-” means no data available in GenBank. The taxa marked with a “T” are type specimens.

**Table 2:** Sequence information and GenBank numbers of samples used in *Pleurocordyceps* study.

Taxon	Strain	ITS	SSU	LSU	*rpb1*	*rpb2*	*tef-1α*	Country
*Perennicordyceps cuboidea*	NBRC 10383	JN943319	JN941735	JN941406	JN992469	AB968563	AB968602	Japan
*Pe*. *cuboidea*	NBRC 101740	JN943321	JN941734	JN941407	JN992468	AB968564	AB968603	Japan
*Pleurocordyceps agarica*	YHCPA1307	KP276654	-	-	KP276666	KP276670	KP276662	China
*P*. *aurantiaca*	GZU 20-2306	OQ172069	-	OQ172041	-	OQ459789	-	Thailand
*P. aurantiaca*	MFLU 17-1394	MG136918	MG136906	MG136912	MG136867	MG136872	MG136876	Thailand
*P. aurantiaca*	MFLU 21-0276	OQ172072	-	OQ172042	-	OQ459788		Thailand
*P. aurantiaca*	MFLUCC 17-2113	MG136916	MG136904	MG136910	MG136866	MG136870	MG136875	Thailand
*P. clavisynnema* T	GZLG 20-102	OQ968788	-	OQ968796	-	-	OQ982009	China
*P. clavisynnema*	GZCC 22-2042	OQ968789	OQ968805	OQ968797	OQ981998	OQ982004	OQ982008	China
*P. formosus*	MFLU 18-0162	MK863250	MK863043	MK863050	-	-	-	China
*P. heilongtanensis*	HKAS:148549	PV701341	PV791134	PV701302	-	-	-	China
*P. heilongtanensis*	HKAS:148554	PV701372	PV702211	PV701332	-	-	-	China
*P. heilongtanensis*	HKAS:148574	PV737571	PV739068	-	-	PV740523	PV740522	China
*P. heilongtanensis*	KUMCC 3008	OQ172091	OQ172111	OQ172063	OQ459759	OQ459805	OQ459731	China
*P. lanceolatus*	GACPCC 17-2005	OQ172077	-	OQ172047	OQ459755	OQ459801	OQ459727	China
*P. lianzhouensis*	GIMYY9603	EU149922	KF226249	KF226250	KF226251	-	KF226252	-
*P. lianzhouensis*	HIMGD20918	EU149921	KF226245	KF226246	KF226247	-	KF226248	-
*P. marginaliradians*	MFLU 17-1582	MG136920	MG136908	MG136914	MG136869	MG271931	MG136878	Thailand
*P. marginaliradians*	MFLUCC 17-2276	MG136921	-	MG136915	-	-	MG136879	Thailand
*P. multisynnema*	GZCC 22-2041	OQ968793	OQ968803	OQ968801	OQ981997	OQ982003	OQ982010	-
*P. multisynnema* T	GZLG 23-101	OQ968792	OQ968802	OQ968800	-	OQ982002	-	China
*P. neoagarica*	GZCC 22-2043	OQ968791	OQ968804	OQ968794	OQ981996	OQ981999	OQ982007	China
*P. neoagarica* T	GZLC23-103	OQ968790	-	OQ968795	-	-	-	China
*P. neoagarica*	HKAS:148550Y	PV701361	PV791136	PV701323	-	-	-	China
*P. nipponica*	HKAS 100058	MG029523	MG725823	MG725825	-	-	-	China
*P. nipponi* *ca*	NHJ 4268	KF049657	KF049621	KF049639	MF416676	KF049676	MF416517	-
*P. nutansis*	GACP 19-1906	OQ172079	OQ172117	OQ172049	OQ459763	OQ459809	OQ459737	China
*P. nutansis* T	MFLU 21-0275	OQ172073	OQ172119	OQ172048	OQ459765	OQ459811	OQ459739	China
*P. onorei*	BRA CR23902	KU898841	-	-	-	-	-	Ecuador
*P. onorei*	BRA CR23904	KU898843	-	-	-	-	-	Ecuador
*P. phaothaiensis*	BCC84557	MF959733	-	MF959737	MF959745	-	MF959742	Thailand
*P* *. puerensis*	GACPY117	PP627512	PP707759	PP616709	PP654216	-	PP654214	China
*P. ramosopulvinat* *a*	EFCC 5566	KF049658	-	KF049627	KF049645	-	KF049682	-
*P. ramosus*	NBRC 101760	MN586827	MN586818	MN586836	MN598042	MN598060	MN598051	-
*P. sinensis*	MFLU 21-0270	-	-	-	OQ459751	OQ459796	-	China
*P. sinensis*	MFLU 21-0271	-	-	-	OQ459752	OQ459797	-	China
*P. sanduensis*	GZCC 22-2044	OQ968787	OQ968806	OQ968799	OQ981995	OQ982001	OQ982006	China
*P. sanduensis* T	GZLG 23-104	OQ968786	-	OQ968798	-	OQ982000	OQ982005	China
***P. tianmushanensis*** T	**SYYCC001**	**PZ070746**	**PZ070751**	**PZ134459**	**PZ124379**	**PZ095765**	**PZ124381**	**China**
***P. tianmushanensis***	**SYYCC002**	**PZ070747**	**PZ070752**	**PZ134460**	**PZ124380**	**PZ095766**	**PZ124382**	**China**
*P. tomentosus*	BL4	KF049666	KF049623	KF049641	KF049656	KF049678	KF049697	-
*P. vitellina*	KUMCC 3005	OQ172088	-	OQ172060	OQ459756	OQ459802	OQ459728	China
*P. vitellina*	KUMCC 3006	OQ172089	-	OQ172061	OQ459757	OQ459803	OQ459729	China
*Pleurocordyceps* sp.	GZU 19-1907	OQ172087	-	-	OQ459764		-	China
*Pleurocordyceps* sp.	GZU 19-3019	OQ172086	-	-	OQ459766	OQ459812	-	China
*Pleurocordyceps* sp.	YFCC 07239279	-	-	-	PP581807	PP581824	PP254877	China
*Pleurocordyceps* sp.	YFCC 07239280	-	-	-	PP581808	PP581825	PP254878	China

Notes: Newly generated sequences are indicated in bold. “-” means no data available in GenBank. The taxa marked with a “T” are type specimens.

**Table 3:** Base pair differences in *Pleurocordyceps tianmushanensis*, *P. sanduensis*, *P. puerensis*, and *P. clavisynnema* with gaps.

Species Pair	ITS	SSU	LSU	*rpb1*	*rpb2*	*tef-1α*
*P. tianmushanensis* vs. *P. sanduensis*	1/607 (0.2%)	7/1082 (0.6%)	0/734	-	4/918 (0.4%)	7/846 (0.8%)
*P. tianmushanensis* vs. *P. puerensis*	4/512 (0.8%)	15/798 (1.9%)	0/716	1/594 (0.2%)	-	5/855 (0.6%)
*P. tianmushanensis* vs. *P. clavisynnema*	1/594 (0.2%)	0/1231	0/856	2/642 (0.3%)	4/968 (0.4%)	4/878 (0.5%)
*P. sanduensis* vs. *P. puerensis*	2/607 (0.3%)	17/701 (0.1%)	0/821	1/672 (0.1%)	-	6/867 (0.7%)
*P. sanduensis* vs. *P. clavisynnema*	0/518	8/1130 (0.3%)	0/815	2/678 (0.3%)	3/1050 (0.3%)	23/824 (2.8%)
*P. puerensis* vs. *P. clavisynnema*	3/552 (0.5%)	0/800	0/764	1/676 (0.1%)	-	7/795 (0.9%)

## Data Availability

The data presented in the study are deposited in the Herbarium of Hangzhou Normal University, accession numbers T587, T5872, SYYCC001, and SYYCC002.

## Abbreviations

The following abbreviations are used in this manuscript. The generic names referenced in this study are abbreviated as follows: *C.* = *Cudonia*, *P.* = *Pleurocordyceps*, *Pe*. = *Perennicordyceps*. In the fungal descriptions, the symbol “x” represents the mean value of the measured characteristic; “n” represents the number of measured objects.

## References

[1] Fries E.M. (1849). Summa Vegetabilium Scandinaviae.

[2] Imai S. (1936). Studies on the Geoglossaceae of Japan. III The genus *Cudonia*. Shokubutsugaku Zasshi.

[3] Lantz H., Johnston P.R., Park D., Minter D.W. (2011). Molecular phylogeny reveals a core clade of Rhytismatales. Mycologia.

[4] Yang Z. (2023). Two new species of *Cudonia* (Rhytismatales) from Southwestern China. Mycosystema.

[5] Durand E.J. (1908). The Geoglossaceae of North America. Ann. Mycol..

[6] Corner E.J.H. (1929). Studies in the morphology of Discomycetes: II. The structure and development of the ascocarp. Trans. Br. Mycol. Soc..

[7] Corner E.J.H. (1930). Studies in the morphology of Discomycetes: III. The Clavuleae. Trans. Br. Mycol. Soc..

[8] Imai S. (1941). Geoglossaceae Japoniae. J. Fac. Agric. Hokkaido Imp. Univ..

[9] Mains E.B. (1940). New and unusual species of the Geoglossaceae. Am. J. Bot..

[10] Mains E.B. (1955). North American hyaline-spored species of the Geoglosseae. Mycologia.

[11] Wang Z.J., Binder M., Schoch C.L., Johnston P.R., Spatafora J.W., Hibbett D.S. (2006). Evolution of helotialean fungi (Leotiomycetes, Pezizomycotina): A nuclear rDNA phylogeny. Mol. Phylogenet. Evol..

[12] Platt J.L., Spatafora J.W. (2000). Evolutionary relationships of nonsexual lichenized fungi: Molecular phylogenetic hypotheses for the genera *Siphula* and *Thamnolia* from SSU and LSU rDNA. Mycologia.

[13] Gargas A., DePriest P.T., Grube M., Tehler A. (1995). Multiple origins of lichen symbioses in fungi suggested by SSU rDNA phylogeny. Science.

[14] Binder M., Hibbett D.S. (2002). A new species of *Cudonia* based on morphological and molecular data. Mycologia.

[15] Burenbaatar G., Chen Z.Q., Bau T. (2025). *Cudonia Mongolica* sp. nov. (Cudoniaceae, Rhytismatales) from Mongolia. Phytotaxa.

[16] Kirk P., Cannon P., David J., Staplers J.A. (2001). Bisby’s Dictionary of the Fungi.

[17] Ge Z.W., Yang Z.L., Pfister D.H., Carbone M., Bau T., Smith M.E. (2014). Multigene molecular phylogeny and biogeographic diversification of the earth tongue fungi in the genera *Cudonia* and *Spathularia* (Rhytismatales, Ascomycota). PLoS ONE.

[18] Wang Y.H., Ban S., Wang W.J., Li Y., Wang K., Kirk P.M., Bushley K.E., Dong C.H., Hawksworth D.L., Yao Y.J. (2021). *Pleurocordyceps* gen. nov. for a clade of fungi previously included in *Polycephalomyces* based on molecular phylogeny and morphology. J. Syst. Evol..

[19] Xiao Y.P., Yang Y., Jayawardena R.S., Gentekaki E., Peng X.C., Luo Z.L., Lu Y.Z. (2024). Four novel *Pleurocordyceps* (Polycephalomycetaceae) species from China. Front. Microbiol..

[20] Dong Q.Y., Zeng N.K., Zhou J.N., Gao S.Y., Xu C.D., Wang Z.J. (2026). Molecular phylogeny and morphology reveal two novel entomopathogenic species of Hypocreales (Polycephalomycetaceae and Cordycipitaceae), from China. MycoKeys.

[21] Xiao Y.P., Wang Y.B., Hyde K.D., Eleni G., Sun J., Yang Y., Meng J., Yu H., Wen T.C. (2023). Polycephalomycetaceae, a new family of Clavicipitoid fungi segregates from Ophiocordycipitaceae. Fungal Divers..

[22] Crous P.W., Wingfield M.J., Burgess T.I., Carnegie A.J., Hardy G.E.S.J., Smith D., Summerell B.A., Cano-Lira J.F., Guarro J., Houbraken J. (2017). Fungal planet description sheets: 625–715. Persoonia.

[23] Poinar G., Vega F.E. (2020). Entomopathogenic fungi (Hypocreales: Ophiocordycipitaceae) infecting bark lice (Psocoptera) in Dominican and Baltic amber. Mycology.

[24] Wang Y., Yu H., Dai Y.D., Chen Z.H., Zeng W.B., Yuan F., Liang Z.Q. (2015). *Polycephalomyces yunnanensis* (Hypocreales), a new species of *Polycephalomyces* parasitizing *Ophiocordyceps nutans* and stink bugs (hemipteran adults). Phytotaxa.

[25] Cao B., Phurbu D., Ralaiveloarisoa A., Liimatainen K., Niskanen T., Ramírez-Cruz V., Bradshaw A.J., Dentinger B.T.M., Ramírez-Guillén F., Cortés-Pérez A. (2025). Fungal diversity notes 1919–2016: Taxonomic and phylogenetic contributions to fungal taxa. Fungal Divers..

[26] Wu R., Zeng X., McCormack M.L., Fernandez C.W., Yang Y., Guo H., Xi M., Liu Y., Qi X., Liang S. (2024). Linking root-associated fungal and bacterial functions to root economics. eLife.

[27] Kornerup A.W.J., Wanscher J.H. (1978). Methuen Handbook of Colour.

[28] Sun J.Z., Liu X.Z., McKenzie E.H.C., Jeewon R., Liu J.K., Zhang X.L., Zhao Q., Hyde K.D. (2019). Fungicolous fungi: Terminology, diversity, distribution, evolution, and species checklist. Fungal Divers..

[29] White T.J. (1990). Amplification and direct sequencing of fungal ribosomal RNA genes for phylogenetics. PCR Protocols, a Guide to Methods and Applications.

[30] Sangdee A., Sangdee K., Seephonkai P., Jaihan P., Kanyaphum T. (2017). Colony characteristics, nucleoside analog profiles, and genetic variations of medicinal fungus *Polycephalomyces nipponicus* (Ascomycetes) isolates from Northeast Thailand. Int. J. Med. Mushrooms.

[31] Sonyot W., Lamlertthon S., Luangsa-ard J.J., Mongkolsamrit S., Usuwanthim K., Ingkaninan K., Waranuch N., Suphrom N. (2020). In vitro antibacterial and anti-Inflammatory effects of novel insect fungus *Polycephalomyces phaothaiensis* extract and its constituents against *Propionibacterium acnes*. Antibiotics.

[32] Vilgalys R., Hester M. (1990). Rapid genetic identification and mapping of enzymatically amplified ribosomal DNA from several *Cryptococcus* species. J. Bacteriol..

[33] Hopple J.S., Vilgalys R. (1999). Phylogenetic relationships in the mushroom genus *Coprinus* and dark-spored allies based on sequence data from the nuclear gene coding for the large ribosomal subunit RNA: Divergent domains, outgroups, and monophyly. Mol. Phylogenet. Evol..

[34] Castlebury L.A., Rossman A.Y., Sung G.H., Hyten A.S., Spatafora J.W. (2004). Multigene phylogeny reveals new lineage for *Stachybotrys chartarum*, the indoor air fungus. Mycol. Res..

[35] Yang Y., Xiao Y., Yu G., Wen T., Deng C., Meng J., Lu Z. (2021). *Ophiocordyceps aphrophoridarum* sp. nov., a new entomopathogenic species from Guizhou, China. Biodivers. Data J..

[36] Nilsson R.H., Tedersoo L., Abarenkov K., Ryberg M., Kristiansson E., Hartmann M., Schoch C.L., Nylander J.A.A., Bergsten J., Porter T.M. (2012). Five simple guidelines for establishing basic authenticity and reliability of newly generated fungal ITS sequences. MycoKeys.

[37] Alzohairy A. (2011). BioEdit: An important software for molecular biology. GERF Bull. Biosci..

[38] Saccardo P.A., Berlese A.N. (1883). Miscellanea Mycologica.

